# Exogenous melatonin reduces somatic cell count of milk in Holstein cows

**DOI:** 10.1038/srep43280

**Published:** 2017-02-27

**Authors:** Minghui Yang, Jianmin Shi, Jianhua Tian, Jingli Tao, Menglong Chai, Jing Wang, Zhiyuan Xu, Yukun Song, Kuanfeng Zhu, Pengyun Ji, Guoshi Liu

**Affiliations:** 1 National Engineering Laboratory for Animal Breeding, Key Laboratory of Animal Genetics and Breeding of the Ministry of Agriculture, Beijing Key Laboratory for Animal Genetic Improvement, College of Animal Science and Technology, China Agricultural University, Beijing, China; 2 National Animal Husbandry Service, Beijing, China

## Abstract

High somatic cell counts in milk caused by mastitis significantly influence the quality of milk and result in substantial annual economic loss. This study evaluated the beneficial effects of melatonin (MT) on milk somatic cell count (SCC) in cows. To examine the effects of melatonin on SCC, one hundred twenty cows were divided into four groups based on milk SCC. In each group, half of the cows were treated with melatonin (S.C.). Melatonin treatment significantly reduced milk SCC. To explore the potential mechanism, 20 cows with relatively high SCC were selected to evaluate the biochemical and immunological profiles of their blood after melatonin treatment. Treatment with MT significantly reduced SCC in milk, lowered serum cortisol concentrations and increased the levels of albumin, alanine transaminase and lactate dehydrogenase. Following treatment with MT, the concentration of IgG and IgM rose transiently then decreased significantly, similar to changes observed for white blood cells and lymphocytes. In conclusion, MT treatment improved the quality of milk by reducing SCC. This may be due to melatonin improving immune activity in cows.

Mastitis is the most common disease in dairy cows^1^. There are two major forms of this disease, including clinical and subclinical mastitises^2^. Clinical mastitis is easily identified by swelling and redness in the teat and udder or increased temperature in the infected mammary gland^3^. However, the subclinical form is difficult to detect because there are no apparent symptoms. It is mainly characterized by an increase in the somatic cell count (SCC) of milk and a decrease in milk production^4^. Animals are considered to have a subclinical mammary gland infection if milk contains SCC > 1 × 10^5^ cells/mL^5^. although some studies have used a threshold of 2 × 10^5^ cells/mL^6^. The extremely high prevalence of subclinical mastitis causes great economic loss in the dairy industry. In China, 40–80% of milking cows have subclinical mammary gland infections, and the annual loss is estimated at 135 million Yuan^7^. Although antibiotic therapy has a direct and rapid effect on pathogenic bacteria, the frequent use of antibiotics leads to drug-resistant bacteria. In addition, drug residue in milk has a negative impact on human health^8^. Therefore, alternative therapies for bovine mastitis are necessary.

Reactive oxygen species (ROS) are continuously formed because of biochemical reactions and external stimulations during the substantial metabolic and physiological adaptations cows undergo between pregnancy and lactation^9^. Excessive ROS production causes oxidative stress in organisms, which is a significant underlying factor in the dysfunction of host immune and inflammatory responses. Thus, oxidative stress in dairy cows inevitably leads to increased susceptibility to a variety of health disorders, particularly during the high-temperature period^10^,^11^,^12^,^13^,^14^. Melatonin (MT), a hormone mainly synthesized in the pineal gland, affects a variety of physiological processes. It is a signalling molecule in circadian rhythms, regulates reproduction for photoperiodic breeders, modulates the neuroendocrine system and provides cardiovascular protection^15^,^16^,^17^,^18^. MT is a potent free radical scavenger and antioxidant^19^,^20^,^21^. In addition to its antioxidant function, MT balances immune responses and can be an immunostimulatory or immunosuppressive agent depending on conditions. For example, during acute inflammation, it exhibits anti-inflammatory effects and suppresses exacerbated immune responses^22^. MT implants in goats improved milk quality and reduced SCC in milk by decreasing oxidative stress in the udder. Reports have linked high nocturnal levels of MT in cows with low milk SCC^23^.

This study investigated the effect of MT administration (S.C.) on the quality of the milk produced by cows with subclinical mastitis. Analyses included SCC in milk and cellular and humoural immune responsive parameters in blood^24^.

## Results

### Effects of melatonin treatment on SCC in milk

**Figure 1:**
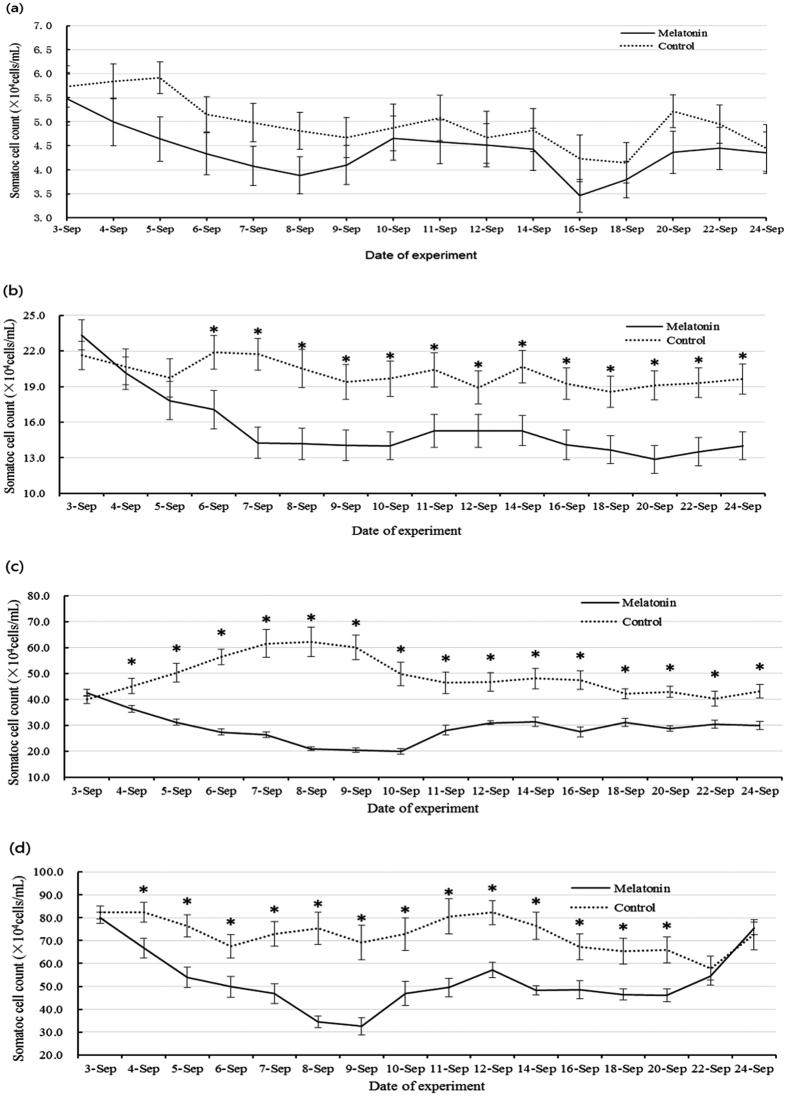
Changes in milk SCC following the subcutaneous injection of melatonin into cows over four consecutive days. (**a**) Cows with milk SCC below 1 × 10^5^ cells/mL; this group served as healthy controls. (**b**) Cows with milk SCC ranging from 1 × 10^5^ to 3 × 10^5^ cells/mL. (**c**) Cows with milk SCC ranging from 3 × 10^5^ to 5 × 10^5^ cells/mL. (**d**) Cows with milk SCC ranging from 5 × 10^5^ to 1 × 10^6^ cells/mL. Melatonin was given on 3-Sep, 4-Sep, 5-Sep and 6-Sep. Data are expressed as the means ± SEM (n = 15). “*” represents significant differences (p < 0.05).

Treatment with MT (4.64 mg/cow/day for 4 days) reduced the milk SCC in all treated groups (Fig. 1), including significant differences between the relatively high SCC groups (groups 2–4) compared with their untreated counterparts. This effect lasted for a minimum of 24 days after the termination of melatonin treatment.

### Effects of melatonin on plasma cortisol levels

The results of MT treatment on cortisol concentrations are listed in Table 1. Plasma cortisol levels of Holstein cows were positively associated with their milk SCC. In cows with subclinical mastitis, cortisol levels were significantly higher (5.93 ± 1.70 to 6.12 ± 1.68 ng/mL) than in healthy cows, (3.42 ± 0.33 ng/mL). After MT treatment, the relatively high cortisol levels in cows with subclinical mastitis were reduced to normal ranges, as observed in the control group.

### Effects of melatonin on blood biochemical indices

The results of MT treatment on blood biochemical parameters, including IgG, IgM, albumin, alanine aminotransferase (ALT) and serum lactate dehydrogenase (S-LDH) are listed in Table 2. IgG and IgM levels in cows with subclinical mastitis (10.26 ± 1.31 to 10.86 ± 1.82 g/L) were significantly higher than in control groups (2.63 ± 0.21 g/L to 2.74 ± 0.63 g/L) (p < 0.05). In contrast, the blood levels of ALB, ALT and LDH were significantly lower in cows with subclinical mastitis than in healthy cows. There were no significant differences in blood glucose levels among these groups (p > 0.05). There was a transient increase in blood IgG and IgM levels in cows with subclinical mastitis one day after the termination of melatonin treatment followed by a significant decrease three days after termination. Following treatment, IgM concentrations were reduced to levels observed in the healthy group. Blood ALB, ALT and LDH levels in cows with subclinical mastitis were significantly increased after melatonin treatment compared with their untreated counterparts (p < 0.05). There were no significant differences in the blood levels of LDH among groups before or after melatonin injection.

### Effects of melatonin on blood cells

The effects of MT treatment on blood cells are listed in Table 3. WBC, LYM and GRN were significantly higher in cows with subclinical mastitis compared with healthy cows (p < 0.05). There were no significant differences in RBC between groups (p > 0.05). In cows with subclinical mastitis treated with melatonin, the number of WBC and LYM changed in a pattern similar to that described for IgG and IgM, there was a transient increase then a decrease compared with untreated counterparts. However, levels were not reduced to the concentrations observed in the healthy group.

## Discussion

SCC in milk is an important index of breast health in cows^25^. To some degree, the quality of milk is determined by its SCC. A significant increase of SCC in milk indicates a poor quality product, with high SCC accompanied by relatively low levels of butterfat, milk protein, calcium and relatively high levels of sodium and chloride. All these factors lower the nutritional value of milk and, in some cases, necessitate discarding the milk. A high SCC in milk is also an important factor for determining whether to cull a cow. Approximately 30% of cows are culled due to high SCC in their milk^26^. When SCC in milk is higher than 7 × 10^5^ cells/mL, the culling rate is 3–4 times higher than in cows with milk SCC lower than 2.5 × 10^5^ cells/mL^27^. In this study, cows with milk SCC greater than 3 × 10^5^ cells/mL were classified as having subclinical mastitis.

ROS, caused by high environmental temperatures, cause immunosuppression in dairy cows, aggravate dairy mastitis and improve SCC^28^. Dairy cow mastitis is connected with the activity of reactive oxygen species, cortisol, cytokines, and other classic inflammatory mediators^29^. The conventional treatment for mastitis in cows is antibiotics, due to their rapid therapeutic effect on mastitis. However, the frequent use of antibiotics causes drug resistance, and drug residues in milk directly jeopardize human health, especially in children. Therefore, it is crucial to find suitable alternative therapies for dairy cow mastitis. Naturally occurring antioxidants have antiviral effects, scavenge reactive oxygen species and improve immune response. They also improve reproductive performance in cows, lower SCC in raw milk and reduce the incidence of mastitis^30^. Dietary supplementation with tea polyphenol effectively reduced the SCC in raw milk^31^. We observed that melatonin, a naturally occurring antioxidant, effectively eliminates ROS and enhances immunity. We also found that a short period (four days) of administration (S.C.) significantly reduced the SCC of milk in Holstein cows with subclinical mastitis. Cortisol plays a pivotal role in inflammatory responses, such as mastitis. It regulates glucose metabolism, which is a blood immune inhibitor, and inhibits immune-activity. Serum cortisol levels increase in stress states and promote protein decomposition and glycogen dysplasia. When cows are stressed, cortisol secretion increases and immune function is repressed, including the atrophy of immune organs and a decrease in number of lymphocytes and acidophilic leukocytes in blood. All of these factors lead to the inhibition of cellular immunity and resistance to disease^32^. This is consistent with our observation that blood cortisol levels in cows with subclinical mastitis (milk SCC > 3 × 10^5^ cells/mL) were significantly higher than in healthy cows (milk SCC < 1 × 10^5^ cells/mL). Melatonin injection significantly reduced cortisol levels in cows with subclinical mastitis, possibly via melatonin-mediated suppression of adrenal cell corticotropin releasing hormone and adrenocorticotropic hormone, which leads to a reduction in butyrylcholinesterase cAMP-induced cortisol production. In addition, the rate-limiting step in cortisol synthesis is the conversion of cholesterol to pregnenolone. Melatonin reduces the synthesis of cortisol by inhibiting prostaglandin^33^. which reduces the concentration of intracellular cAMP in lymphocytes.

IgG and IgM are important factors in immune response and anti-infection. They lyse and activate complements to promote phagocytosis. Research shows that melatonin enhances humoural immunity^34^. In mice treated with inactivated Venezuelan equine encephalomyelitis virus, subcutaneous injection of melatonin significantly increased serum globulin and IgM levels^35^. Melatonin administration also enhanced the levels of IgG and IgM in 28-month-old Wistar rats^36^. We observed that melatonin treatment transiently increased IgG and IgM levels in cows with subclinical mastitis before levels were reduced toward normal. This implies that melatonin treatment decreases symptoms of mastitis while it decreases IgG and IgM levels, consistent with our observation that melatonin treatment decreases SCC in milk. Lymphocytes and neutrophils are also important factors in inflammatory and immune responses. Studies show that extending the illumination time at night decreases the percentage of peripheral lymphocytes, which also suppresses the secretion of melatonin in animals. However, treatment with melatonin significantly increased the percentage of lymphocytes^37^,^38^. In pinealectomized animals, thymus and spleen function were suppressed, as indicated by a decrease in the number of lymphocytes and neutrophils. Treatment with melatonin led to a full recovery. These studies are consistent with our observation that melatonin injection significantly enhanced the number of lymphocytes in cows with high milk SCC.

## Conclusion

We report, for the first time, that subcutaneous injection of melatonin significantly reduces SCC in the milk of cows with subclinical mastitis. The mechanism of action may be related to the antioxidant, anti-inflammatory and immunoenhancement activities of melatonin. For cows with mastitis, the administration of melatonin reduced cortisol levels and upregulated levels of IgG, IgM, lymphocytes and neutrophils. Melatonin is a naturally occurring antioxidant with low to no toxicity that is inexpensive and widely available. SCC in milk directly reflects the quality of the milk and affects the health of consumers. Treatment with melatonin may provide an effective, safe and quick method to lower SCC in milk and replace the use of the antibiotics, which have many potential adverse effects for cows and human health. To understand the exact mechanisms of melatonin’s beneficial effects on cows with high levels of SCC in their milk, additional studies are necessary.

## Materials and Methods

### Ethics Statement

This study was carried out in strict accordance with the guidelines for the care and use of animals of China Agricultural University. All animal experimental procedures were approved by the Animal Care Commission of the College of Animal Science and Technology, China Agricultural University. Every effort was made to minimize animal pain, suffering, and distress and to reduce the number of animals used.

### Management of animals

Studies were performed in a commercial dairy herd in Beijing China. One hundred forty Holstein dairy cows were included in the experiment. The approximate annual milk production was 9,102 to 11,020 kg per cow for the herd during this study period. Cows were milked three times per day and fed with TR diet (NRC2001). The mean annual culling rate for the study period was 29.5%. Lactating cows were reared in cubicle and feeding areas. In the feeding area, water sprinklers facing the resting place of cows were automatically activated when the temperature reached approximately 25 °C.

### Experimental design

#### Experiment I

One hundred twenty cows were divided into four groups (<10 × 10^4^ cells/mL, 1 × 10^5^ to 3 × 10^5^ cells/mL, 3 × 10^5^ to 5 × 10^5^ cells/mL and 5 × 10^5^ to 1 × 10^6^ cells/mL, respectively) depending on milk SCC levels. Each group was randomly subdivided into melatonin treated and untreated cows. Melatonin treated cows were given subcutaneous injections of MT (4.64 mg) for four consecutive days. Milk was sampled to examine the effects of melatonin on milk SCC.

#### Experiment II

Twenty cows with milk SCC in the range of 3 × 10^5^ to 1 × 10^6^ cells/mL were selected. Half were treated with MT (4.64 mg) for four consecutive days and the remainder served as untreated controls. This experiment was designed to find associations between melatonin treatment, milk SCC and alterations in the biochemical and immunoresponsive parameters in blood. Thus, both milk and blood were sampled from cows at different time points.

### Melatonin injection

Melatonin (Sigma-Aldrich Chemical Co. St. Louis, MO, USA.) was dissolved in ethanol and diluted with normal saline while in a darkroom. Each animal was given 4.64 mg melatonin in a parenteral solution. Cows were injected subcutaneously in the neck with melatonin for four consecutive days at 8:00 am.

### Blood sampling

Blood samples were drawn from the coccygeal blood vessel of each cow just before the first melatonin injection at 8:00 am. Thereafter, blood samples were collected at 8:00 am on days one and three after the last melatonin injection. Blood samples were collected in the vacutainers containing heparin or EDTA for analyses of cortisol, the number of red blood cells, lymphocytes, and neutrophils and measurement of physiological and biochemical indices, such as IgG, IgM, albumin and alanine transaminase and lactate dehydrogenase. Samples were immediately placed on ice, and plasma was separated by centrifugation within 1 h of collection. Measurements of serum metabolites and routine blood tests were conducted at the laboratories of BJ. XinChuangYuan BIOTECH CO., LTD (Beijing, China).

### Milk sampling

Milk was sampled each day for three days before melatonin treatment and then sampled for 18 days after the last melatonin injection. Milk samples were used for analysis of SCC. All samples were collected after morning milking. The milk samples were heated to 40–42 °C. Samples were well shaken, then analysed using a fluorescence optical system (Fossomatic TM Minor; FossElectric; Hillerød; Denmark).

### Statistical analysis

Data, expressed as the mean ± SEM, were analysed with univariate analysis of variance (ANOVA) followed by Duncan’s test using SPSS 19.0 statistical software. The significant difference between treatments was set at P < 0.05.

## Additional Information

**How to cite this article:** Yang, M. *et al*. Exogenous melatonin reduces somatic cell count of milk in Holstein cows. *Sci. Rep.* **7**, 43280; doi: 10.1038/srep43280 (2017).

**Publisher's note:** Springer Nature remains neutral with regard to jurisdictional claims in published maps and institutional affiliations.

## Figures and Tables

**Table 1:** Effects of subcutaneous injection of melatonin on plasma cortisol in Holstein cows (ng/mL).

Group	Days after treatment		
	0	1	3
MT (SCC 3 × 10^5^1 × 10^6^ cells/mL)	6.12 ± 1.68^Aa^	3.43 ± 1.11^Bb^	3.12 ± 0.56^Bb^
NON-MT (SCC 3 × 10^5^–1 × 10^6^ cells/mL)	5.93 ± 1.70^Aa^	5.26 ± 1.13^Aa^	5.82 ± 1.53^Aa^
Healthy control (SCC < 1 × 10^5^ cells/mL)	3.42 ± 0.33^b^	—	—

MT (melatonin-treated cows); NON-MT (non-melatonin-treated cows). Data expressed as the means ± SEM (n = 20). Values with different superscript letters, such as^ab^, represent a significant difference within the same group, (P < 0.05); values with different superscript letters^AB^ represent a significant difference between different groups (P < 0.05); the same letters represent no significant difference (P > 0.05).

**Table 2:** Effects of subcutaneous injection of melatonin on blood biochemical index of Holstein cows.

Group	Index	Before injection	1 d after injection	3 d after injection
MT (SCC:3 × 10^5^–1 × 10^6^ cells/mL)	IgG (g/L)	10.26 ± 1.31^Aa^	12.18 ± 1.26^Ba^	8.92 ± 0.63^Ca^
NON-MT (SCC:3 × 10^5^–1 × 10^6^ cells/mL)		10.86 ± 1.82^Aa^	10.53 ± 1.35^Ab^	10.29 ± 1.27^Ab^
Healthy control (SCC < 1 × 10^5^ cells/mL)		5.87 ± 1.01^c^	—	—
MT (SCC:3 × 10^5^–1 × 10^6^ cells/mL)	IgM (g/L)	2.63 ± 0.21^Aa^	3.39 ± 0.23^Ba^	2.14 ± 0.20^Aa^
NON-MT (SCC:3 × 10^5^–1 × 10^6^ cells/mL)		2.74 ± 0.63^Aa^	2.75 ± 0.44^Ab^	2.65 ± 0.44^Ab^
Healthy control (SCC < 1 × 10^5^ cells/mL)		2.12 ± 0.20^b^	—	—
MT (SCC:3 × 10^5^–1 × 10^6^ cells/mL)	ALB (g/L)	29.92 ± 1.44^Aa^	33.58 ± 1.31^Ba^	33.92 ± 1.44^Ba^
NON-MT (SCC:3 × 10^5^–1 × 10^6^ cells/mL)		30.08 ± 1.78^Aa^	31.75 ± 1.60^Ab^	28.25 ± 1.22^Bb^
Healthy control (SCC < 1 × 10^5^ cells/mL)		38.5 ± 2.72^c^	—	—
MT (SCC:3 × 10^5^–1 × 10^6^ cells/mL)	ALT (U/L)	25.42 ± 4.03^Aa^	34.17 ± 4.04^Ba^	35.50 ± 4.38^Ba^
NON-MT (SCC:3 × 10^5^–1 × 10^6^ cells/mL)		24.83 ± 3.93^Aa^	30.00 ± 4.81^Bb^	31.42 ± 5.95^Bb^
Healthy control (SCC < 1 × 10^5^ cells/mL)		35.5 ± 6.42^b^	—	—
MT (SCC:3 × 10^5^–1 × 10^6^ cells/mL)	LDH (U/L)	801.33 ± 66.48^Aa^	940.67 ± 103.13^Ba^	904.67 ± 96.38^Ba^
NON-MT (SCC:3 × 10^5^–1 × 10^6^ cells/mL)		847.92 ± 85.03^Aa^	844.83 ± 86.80^Ab^	842.83 ± 93.57^Ab^
Healthy control (SCC < 1 × 10^5^ cells/mL)		945.7 ± 87.28^b^	—	—
MT (SCC:3 × 10^5^–1 × 10^6^ cells/mL)	GLU (mmol/L)	4.06 ± 0.23^Aa^	4.32 ± 0.24^Aa^	4.21 ± 0.19^Aa^
NON-MT (SCC:3 × 10^5^–1 × 10^6^ cells/mL)		3.96 ± 0.23^Aa^	4.16 ± 0.31^Aa^	4.14 ± 0.23^Aa^
Healthy control (SCC < 1 × 10^5^ cells/mL)		4.16 ± 0.22^a^	—	—

MT (melatonin-treated cows); NON-MT (non-melatonin-treated cows). Data expressed as the means ± SEM (n = 10). Values with different superscript letters, such as ^ab^represent a significant difference within the same group, (P < 0.05); Values with different superscript letters ^AB^represent a significant difference between different groups (P < 0.05); The same letters represent no significant difference (P > 0.05).

**Table 3:** Effects of subcutaneous injection of melatonin on white and red blood cell counts of Holstein cows.

Group	Index	Before injection	1 d after injection	3 d after injection
MT (SCC:3 × 10^5^–1 × 10^6^ cells/mL)	WBC (*10^9^ cells/L)	13.74 ± 2.63^Aa^	16.58 ± 3.95^Ba^	10.93 ± 2.35^Ca^
NON-MT (SCC:3 × 10^5^–1 × 10^6^ cells/mL)		13.98 ± 6.29^Aa^	14.96 ± 7.50^Aa^	16.35 ± 5.44^Aa^
Healthy control (SCC < 1 × 10^5^ cells/mL)		8.86 ± 1.18^b^	—	—
MT (SCC:3 × 10^5^–1 × 10^6^ cells/mL)	LYM (*10^9^ cells/L)	7.93 ± 4.43^Aa^	13.63 ± 5.92^Aa^	6.14 ± 1.48^Ba^
NON-MT (SCC:3 × 10^5^–1 × 10^6^ cells/mL)		8.62 ± 3.68^Aa^	8.79 ± 2.59^Ab^	12.98 ± 5.40^Ba^
Healthy control (SCC < 1 × 10^5^ cells/mL)		4.75 ± 1.49^b^	—	—
MT (SCC:3 × 10^5^–1 × 10^6^ cells/mL)	GRN (*10^9^ cells/L)	4.35 ± 0.79^Aa^	4.33 ± 0.93^Aa^	3.68 ± 0.83^Aa^
NON-MT (SCC:3 × 10^5^–1 × 10^6^ cells/mL)		4.29 ± 0.65^Aa^	4.64 ± 0.39^Ab^	4.28 ± 0.94^Aa^
Healthy control (SCC < 1 × 10^5^ cells/mL)		3.04 ± 0.51^b^	—	—
MT (SCC:3 × 10^5^–1 × 10^6^ cells/mL)	RBC (*10^12^ cells/L)	5.69 ± 0.40^Aa^	5.61 ± 0.37^Aa^	5.73 ± 0.54^Aa^
NON-MT (SCC:3 × 10^5^–1 × 10^6^ cells/mL)		6.04 ± 0.47^Aa^	5.96 ± 0.50^Aa^	5.86 ± 0.43^Aa^
Healthy control (SCC < 1 × 10^5^ cells/mL)		5.65 ± 0.54^a^	—	—

MT (melatonin-treated cows); NON-MT (non-melatonin-treated cows); WBC: white blood cells; LYM: lymphocytes; GRN: granulocyte neutrophil; RBC: red blood cells. Data expressed as the means ± SEM (n = 10). Values with different superscript letters, such as ^ab^represent a significant difference within the same group, (P < 0.05); values with different superscript letters ^AB^represent a significant difference between different groups, (P < 0.05); the same letters represent no significant difference (P > 0.05).

## References

[b1] Bannerman D. D. Pathogen-dependent induction of cytokines and other soluble inflammatory mediators during intramammary infection of dairy cows. J. Anim. Sci. 13, 10–25 (2009). 10.2527/jas.2008-1187 18708595

[b2] Viguier Caroline, Arora Sushrut, Gilmartin Niamh, Welbeck Katherine & O Kennedy Richard. Mastitis detection: Current trends and future perspectives. Trends Biotechnol. 8, 486–493 (2009). 10.1016/j.tibtech.2009.05.004 19616330

[b3] Wu Junqiang, Hu Songhua & Cao Liting. Therapeutic effect of nisin Z on subclinical mastitis in lactating cows. Antimicrob. Agents Ch. 9, 3131–3135 (2007). 10.1128/AAC.00629-07 PMC2043217 17606675

[b4] Schukken Y. H. . Subclinical and clinical mastitis on dairy farms in the Netherlands: Epidemiological developments. Tijdschr. Diergeneesk. 7, 208 (1995). 7725301

[b5] Kivaria F. M., Noordhuizen J. P. T. M. & Nielen M. Interpretation of California mastitis test scores using Staphylococcus aureus culture results for screening of subclinical mastitis in low yielding smallholder dairy cows in the Dar es Salaam region of Tanzania. Prev. Vet. Med. 3, 274–285 (2007). 10.1016/j.prevetmed.2006.10.011 17137660

[b6] Idriss Sharaf Eldeen Abu Baker . Relationship between mastitis causative pathogens and somatic cell counts in dairy cows. Potravinarstvo. 1, 207–212 (2013).

[b7] Memon J., Javed J. . Molecular characterization and antimicrobial sensitivity of pathogens from sub-clinical and clinical mastitis in Eastern China. Pak. Vet. J. 33, 170–174 (2012).

[b8] Cao L. T., Wu J. Q., Xie F., Hu S. H. & Mo Y. Efficacy of nisin in treatment of clinical mastitis in lactating dairy cows. J. Dairy Sci. 8, 3980–3985 (2007). 10.3168/jds.2007-0153 17639009

[b9] Campbell M. H. & Miller J. K. Effect of supplemental dietary vitamin E and zinc on reproductive performance of dairy cows and heifers fed excess iron. J. Dairy Sci. 10, 2693–2699 (1998). 10.3168/jds.S0022-0302(98)75826-6 9812274

[b10] Allison R. D. & Laven R. A. Effect of vitamin E supplementation on the health and fertility of dairy cows: A review. Veterinary Record. 147, 703–708 (2000). 11140928

[b11] Bernabucci Umberto, Ronchi Bruno, Lacetera Nicola & Nardone Alessandro. Influence of body condition score on relationships between metabolic status and oxidative stress in periparturient dairy cows. J. Dairy Sci. 6, 2017–2026 (2005). 10.3168/jds.S0022-0302(05)72878-2 15905432

[b12] Castillo C. . Oxidative status during late pregnancy and early lactation in dairy cows. The Veterinary Journal 2, 286–292 (2005). 10.1016/j.tvjl.2004.02.001 15727923

[b13] Sordillo Lorraine M. Factors affecting mammary gland immunity and mastitis susceptibility. Livestock Production Science 1, 89–99 (2005).

[b14] Wilde D. Influence of macro and micro minerals in the peri-parturient period on fertility in dairy cattle. Anim. Reprod. Sci. 3, 240–249 (2006). 10.1016/j.anireprosci.2006.08.004 16971071

[b15] Gillette Martha U. & Tischkau Shelley A. Suprachiasmatic nucleus: The brain’s circadian clock. Recent progress in hormone research. 54, 33–60 (1999). 10548871

[b16] Reiter Russel J. Pineal melatonin: Cell biology of its synthesis and of its physiological interactions. Endocr. Rev. 2, 151–180 (1991). 10.1210/edrv-12-2-151 1649044

[b17] Cardinali Daniel P. & Pévet Paul. Basic aspects of melatonin action. Sleep Med. Rev. 3, 175–190 (1998). 10.1016/s1087-0792(98)90020-x 15310500

[b18] Reiter Russel J., Tan Dan-Xian & Korkmaz Ahmet. The circadian melatonin rhythm and its modulation: Possible impact on hypertension. J. Hypertens. 27, 17–20 (2009). 10.1097/01.hjh.0000358832.41181.bf 19633446

[b19] Gitto Eloisa, Pellegrino Salvatore, Gitto Placido, Barberi Ignazio. & Reiter Russel J. Oxidative stress of the newborn in the pre‐and postnatal period and the clinical utility of melatonin. J. Pineal Res. 2, 128–139 (2009). 10.1111/j.1600-079X.2008.00649.x 19054296

[b20] Royano Sergio Damian Paredes & Reiter Russel J. Melatonin: Helping cells cope with oxidative disaster. Cell Membranes and Free Radical Research. 3, 99–111 (2010).

[b21] Reiter R. J., Tan D. X., Manchester L. C. & Tamura H. Melatonin defeats neurally-derived free radicals. J. Physiol. Pharmacol. 6, 5–22 (2007). 18212398

[b22] Carrillo-Vico Antonio, Lardone Patricia J., Álvarez-Sánchez Nuria, Rodríguez-Rodríguez Ana & Guerrero Juan M. Melatonin: Buffering the immune system. Int. J. Mol. Sci. 4, 8638–8683 (2013). 10.3390/ijms14048638 PMC3645767 23609496

[b23] Asher A. . Chrono-functional milk”: The difference between melatonin concentrations in night-milk versus day-milk under different night illumination conditions. Chronobiol. Int. 10, 1409–1416 (2015). 10.3109/07420528.2015.1102149 26588495

[b24] Gellrich Katharina, Sigl Tanja, Meyer Heinrich HD & Wiedemann Steffi. Cortisol levels in skimmed milk during the first 22 weeks of lactation and response to short-term metabolic stress and lameness in dairy cows. Journal of animal science and biotechnology 6, 1–7 (2015). 26244091 10.1186/s40104-015-0035-y PMC4524505

[b25] Caraviello D. Z., Weigel K. A., Shook G. E. & Ruegg P. L. Assessment of the impact of somatic cell count on functional longevity in Holstein and Jersey cattle using survival analysis methodology. J. Dairy Sci. 2, 804–811 (2005). 10.3168/jds.S0022-0302(05)72745-4 15653548

[b26] Gonzalo C., Martínez J. R., Carriedo J. A. & San Primitivo F. Fossomatic cell-counting on ewe milk: Comparison with direct microscopy and study of variation factors. J. Dairy Sci. 1, 138–145 (2003). 10.3168/jds.s0022-0302(03)73593-0 12613858

[b27] Shitandi Anakalo & Kihumbu Gathoni. Assessment of the California mastitis test usage in smallholder dairy herds and risk of violative antimicrobial residues. J. Vet. Sci. 1, 5–10 (2004). 15028880

[b28] Lecchi C., Rota N., Vitali A., Ceciliani F. & Lacetera N. *In vitro* assessment of the effects of temperature on phagocytosis, reactive oxygen species production and apoptosis in bovine polymorphonuclear cells. Vet. Immunol. Immunopathol. 182, 89–94 (2016). 27863557 10.1016/j.vetimm.2016.10.007

[b29] Malinowski E. & Gajewski Z. Mastitis and fertility disorders in cows. J. Vet. Sci. 13, 555–560 (2010). 21033574

[b30] Tezuka M., Suzuki H., Suzuki Y., Hara Y. & Okada S. Inactivation effect of tea leaf catechins on human type-A influenza virus. Japanese Journal of Toxicology and Environmental Health 5, 311–315 (1997).

[b31] Liu S. J., Wang Z. J., Zhang X. K. & Yuan M. Y. Effect of Dietary Biologic Antioxidant Supplementation on Somatic Cell Count of Raw Milk. J. Dairy Sci. 143, 179–182 (2010).

[b32] Du Preez J. H. Parameters for the determination and evaluation of heat stress in dairy cattle in South Africa. Onderstepoort Journal of Veterinary Research 67, 263–271 (2000). 11206394

[b33] Torres Farfan Claudia . Maternal melatonin selectively inhibits cortisol production in the primate fetal adrenal gland. The Journal of physiology 3, 841–856 (2004). 10.1113/jphysiol.2003.056465 PMC1664788 14673186

[b34] Maestroni Georges J. M. The immunotherapeutic potential of melatonin. Expert Opin. Inv. Drug 3, 467–476 (2001). 10.1517/13543784.10.3.467 11227046

[b35] Negrette Beatriz . Melatonin treatment enhances the efficiency of mice immunization with Venezuelan equine encephalomyelitis virus TC-83. Neurochem. Res. 7, 767–770 (2001). 10.1023/a:1011645400123 11565607

[b36] Akbulut K. G., Akbulut K. Gonca., Gönül B. & Akbulut H. The effects of melatonin on humoral immune responses of young and aged rats. Immunol. Invest. 1, 17–20 (2001). 10.1081/imm-100103687 11419908

[b37] Haldar C. & Singh Rajesh. Pineal modulation of thymus and immune function in a seasonally breeding tropical rodent, Funambulus pennanti. Journal of Experimental Zoology 2, 90–98 (2001). 10.1002/1097-010x(20010201)289:2<90::aid-jez2>3.0.co;2-s 11169496

[b38] Singh Shiv Shankar, Haldar, Chandana & Rai, Seema. Melatonin and differential effect of L-thyroxine on immune system of Indian tropical bird Perdicula asiatica. Gen. Comp. Endocr. 3, 215–221 (2006). 10.1016/j.ygcen.2005.09.007 16243326

